# The Edinburgh Cognitive and Behavioural ALS Screen in a Chinese Amyotrophic Lateral Sclerosis Population

**DOI:** 10.1371/journal.pone.0155496

**Published:** 2016-05-19

**Authors:** Shan Ye, Ying Ji, Chengyu Li, Ji He, Xiaolu Liu, Dongsheng Fan

**Affiliations:** Department of Neurology, Peking University Third Hospital, Beijing, China; Center of Genomic & Post Genomics, ITALY

## Abstract

**Objective:**

The existing screening batteries assessing multiple neuropsychological functions are not specific to amyotrophic lateral sclerosis (ALS) patients and are limited to their physical dysfunctions, whereas category cognitive tests are too time-consuming to assess all the domains. The Edinburgh Cognitive and Behavioural ALS Screen (ECAS) was recently developed as a fast and easy cognitive screening tool specifically designed for patients. The purpose of the study was to validate the effectiveness of the Chinese version in Chinese ALS populations.

**Methods:**

Eighty-four ALS patients and 84 age-, gender- and education-matched healthy controls were included in this cross-sectional study. All the participants took the ECAS, Mini-Mental State Examination (MMSE) and Frontal Assessment Battery (FAB). Primary caregivers of patients were interviewed for behavioural and psychiatric changes.

**Results:**

Significant differences were noted in language (p = 0.01), fluency, executive function, ALS-specific functions, and ECAS total score (p<0.01) between ALS patients and controls. The cut-off value of the total ECAS score was 81.92. Cognitive impairment was observed in 35.71% of patients, and 27.38% exhibited behavioural abnormalities. The ECAS total score had a medium correlation with education year. Memory was more easily impaired in the lower education group, whereas verbal fluency and language function tended to be preserved in the higher education group. The average time of ECAS was only 18 minutes.

**Conclusion:**

The Chinese version of the ECAS is the first screening battery assessing multiple neuropsychological functions specially designed for the ALS population in China, which provides an effective and rapid tool to screen cognitive and behavioural impairments.

## Introduction

Amyotrophic lateral sclerosis (ALS) is a multiple-system neurodegenerative disease, and its extra-motor disorders have received increasing concern in recent years. Frontotemporal dementia (FTD) has been known to overlap with ALS. Strong et al. proposed the concept of ALS cognitive impairment (ALS-ci) and behaviour impairment (ALS-bi) [1]. Researchers subsequently found that 10 to 60% of patients had the diagnosis of ALS-ci [2]. Executive difficulty is a typical ALS cognitive domain of which we are familiar [3]. However, non-executive cognitive dysfunctions are also present in ALS [4–6]. These dysfunctions include language, memory and some cases of psychiatric change [7–11]. Thus, different scales are necessary to conduct a comprehensive assessment. Some groups have already undergone screening [12,13], and some measurements have been created [14–20]. However, a single domain cannot reflect the entire profile of cognitive and behavioural change. It is too time-consuming to assess all the domains. Thus, a screening battery assessing multiple neuropsychological functions is needed. Unfortunately, classical measurements, such as the Mini-Mental State Examination (MMSE) [21] and Montreal Cognitive Assessment (MOCA) [22], are not sensitive for the ALS population and have significant limitations for those with physical dysfunctions.

The Edinburgh Cognitive and Behavioural ALS Screen (ECAS) [23] is a brief assessment tool that was designed specifically for ALS patients. The screen includes ALS-specific functions (language, verbal fluency and executive functions) and ALS-non-specific functions (memory and visuospatial functions). Furthermore, primary caregivers completed a questionnaire for five-domain characteristic behavioural changes of FTD and three psychotic questions. The ECAS provides a rapid measurement of cognitive and behavioural functions for ALS, which is not influenced by physical disorders and reflects the severity and nature of the disease. Abrahams’s group later validated the ECAS against a gold standard extensive neuropsychology assessment and demonstrated that the ECAS was a screening tool with high sensitivity and specificity characteristic ALS impairments [24]. The German/Swiss-German version also demonstrated that the ECAS was a fast and easy cognitive screening instrument that was sensitive for ALS-specific dysfunctions and behavioural changes [25].

Regarding the Chinese population, cognitive changes are obviously different between cultures and languages [26]. However, limited assessments have been made in Chinese ALS patients. Yuan et al. investigated the cognition function in 22 early phase patients [27]. Wei et al. [28] screened 145 patients using the MMSE and the revised Addenbrooke’s Cognitive Examination (ACE-R). Cui et al. [29] conducted a neuropsychological investigation between ALS and progressive muscular atrophy (PMA) patients. However, the measurement tools used were time-consuming and potentially restricted by physical dysfunction. Thus, a rapid assessment tool designed especially for Chinese ALS patients is urgent.

The purpose of this research is to validate the Chinese translation of the ECAS as an effective measurement for the Chinese ALS population.

## Materials and Methods

### Participants

We enrolled 84 patients who met the ALS revised El Escorial criteria in the Peking University Third Hospital in 2015 [30]. We excluded patients with the clinical diagnosis of dementia according to ICD-10 criteria as well as those who were illiterate and could not write. As controls, we enrolled 84 healthy people without central nerve system disease who were literate and had the ability to write. No significant differences were noted between patients and healthy controls in terms of age, gender and education (Table 1).

**Table 1:** Characteristics of participants: ALS patients and healthy controls.

Characteristics		ALS patients	Healthy controls
Age (years)		55.07(10.74)33-80	54.83(10.98)31-79
Gender	Female	26	27
	Male	58	57
Years of education		11.45(3.44)6-19	11.51(3.36)6-19
Duration of illness (months)		15.81(14.53)10-78	
ALSFRS-R		41.33(4.64)23-48	
Onset of disease	Bulbar	16(19.05%)	
	Cervical	49(58.33%)	
	Thoracic	3(3.57%)	
	Lumbar	16(19.05%)	
Diagnostic level	Definite	21(25.00%)	
	Probable	25(29.76%)	
	Lab-supported probable	26(30.95%)	
	Possible	12(14.29%)	
Bulbar involved		41(48.81%)	
Ventilation		1(1.19%)	
PEG		0	

Mean (SD) and range are reported for age, gender, year of education, duration of illness, ALSFRS-R. Patient numbers (percentage) are reported for onset of disease, diagnostic level, bulbar involved, ventilation and PEG. There are no significant differences between patients and healthy controls in terms of age, gender and education.ALSFRS-R: Revised ALS Functional Rating Scale. PEG: Percutaneous Endoscopic Gastrostomy.

Primary caregivers of all the patients were interviewed for behavioural and psychiatric changes.

All of the interviews were performed by clinical neurologists. The total interview lasted approximately 25 to 50 minutes, depending on the physical state of the participants.

This study was approved by the Research Ethics Committee of Peking University Third Hospital. All patients were included after informed written consent obtained from patients or their guardians and controls were informed written consent by themselves, as set forth by the Declaration of Helsinki. The consent procedure was approved by ethics committees.

### The Edinburgh Cognitive and Behavioural ALS Screen (ECAS) Chinese version

We translated the Chinese version from the ECAS English version after the authors granted permission, and performed a back-translation by another clinical doctor of neurology who never read the original English version before. Although there were language and cultural differences, we attempted to maintain consistency with the original document. The ECAS Chinese version still contained ALS-specific functions, including language, fluency and executive sections, and ALS-non-specific functions, including memory and visuospatial sections. In addition, five domains of FTD and three domains of psychotic changes were included to interview caregivers in a behavioural screen.

The amendment in the Chinese version compared to the original English version is listed as Table 2. During the translation, two pictures in the “naming” section and the story in the “memory” section were altered according to Chinese culture. The characters chosen for the “spelling” section followed these principles: 1) different structures: left-right structure (跨kua4, 增zeng1, 秽hui4, 短duan3, 颁ban1), top-down structure (管guan3, 焚fen2, 藏cang2), and surrounding structure (廉lian2, 固gu4, 闻wen2, 囵lun2); 2) different component: one part’s pronunciation is the same as the character (夸kua-跨kua, 官guan-管guan, 古gu-固gu, 仑lun-囵lun); one part’s pronunciation is similar to the character but not the same: (曾ceng-增zeng, 岁sui-秽hui, 臧zang-藏cang, 兼jian-廉lian); the character gets its meaning from two parts but not the pronunciation [闻(门door-耳ear-闻hear), 颁(分give-页head-颁give), 焚(木wood-火fire-焚burn), 短(矢measurement unit-豆bean pod-短short)]; 3) frequency of character: high frequency (管0.6194, 增0.3473, 固0.2008, 藏0.1217), middle frequency (短0.02063, 闻0.0089, 跨0.00376, 廉0.00149, 颁0.00144), low frequency (秽0.00044, 焚0.00044, 囵0.00006). The number was the frequency of characters according to the Modern Chinese Frequency Dictionary [31]. Verbal fluency scores were normalized according to the performance of 40 healthy subjects in the preliminary experiment (see Abrahams et al. [23] and guidelines published https://www.era.lib.ed.ac.uk/handle/1842/6592). “发fa1, 开kai1” were chosen in the Chinese version. For the “alternation” section, we chose twelve well-known Chinese zodiac signs. However, except for the two examples of “1-rat, 2-cattle law”, only 10 animals remained. Thus, the total score for this part could only be 10. An additional 2 scores were added to the “sentence completion” section. Thus, the total score for the executive function was 48.

**Table 2:** Amendments in the Chinese version compared to the original English version.

Section	English version	Score	Chinese version	Score
Naming	Scorpion and fox	8	Change into centipede and owl	8
Comprehension	Do not change	8	Do not change	8
Spelling	English words	12	Chinese words	12
Verbal fluency	Letter "T" and "S"	24	Character "fa" and "kai"	24
Reverse digit span	Do not change	12	Do not change	12
Alternation	letters	12	Change into Chinese zodiac signs	10
Sentence completion	6 English sentences	12	7 Chinese sentences	14
Social cognition	Do not change	12	Do not change	12
Immediate recall	English story	10	Chinese story	10
Delayed retention	English story	10	Chinese story	10
Numberlocation	English story	4	Chinese story	4
Dot counting	Do not change	4	Do not change	4
Cube counting	Do not change	4	Do not change	4
Verbal fluency	Do not change	4	Do not change	4
Language		28		28
Verbal fluency		24		24
Executive		48		48
Memory		24		24
Visuospatial		12		12
ALS-specific functions		100		100
ALS-non-specific functions		36		36
Total ECAS score		136		136

The table shows a comparison of each single section and score between the Chinese version and English version.

The ECAS Chinese version is available from the author.

### Other neuropsychological tests

In addition to the Chinese version of the ECAS, we also chose a general cognitive scale, the MMSE [21] and Frontal Assessment Battery (FAB) [32], which is specifically used to assess frontal functions.

### Statistics

Kolmogorov-Smirnov test was used for analyzing normal distribution. To compare clinical features (age and education), score of each section between patients and controls, total ECAS score between different sex and whether bulbar involved, two sided t-test was used if the variables were normally distributed and Mann Whitney U-test was used if not. One-way ANOVA was used to compare total ECAS score between different diagnostic levels and sites of onset. Categorical variable (sex between patients and controls) was compared using a chi-square test. To analyse relationship between the scores of ECAS and other neuropsychological tests, ECAS total score and clinical features (education, age, ALSFRS-R and duration of illness), correlation coefficients were determined via Pearson correlation analysis when the data were continuous, normally distributed and had a linear relationship. Otherwise, Spearman correlation analysis was performed. Cut-off scores were defined as two standard deviations below the mean of the healthy controls according to the work of Abrahams[23]. A Cronbach’s alpha test was used to evaluate reliability. A threshold of p<0.05 was used for statistical inference. Statistical analysis was performed using SPSS 18.0 statistical software.

## Results

### Comparison between patients and controls

We identified significant differences in language, fluency, executive function, ALS-specific functions, and the total ECAS score between ALS patients and controls, whereas no differences in memory, visuospatial and ALS-non-specific functions were noted. However memory (p = 0.07) and ALS-non-specific functions (p = 0.06) seem to have trend to be significant. The mean time of the ECAS was only 18 minutes (Table 3).

**Table 3:** Performances in cognitive scores and ECAS time of ALS patients and healthy controls.

Characteristics	ALS patients	Healthy controls	p
Language	20.01(3.94)9-28	21.71(3.06)13-27	0.01
Fluency	15.28(6.13)0-24	19.44(3.98)2-24	<0.01*
Executive	25.17(8.34)7-46	37.53(6.26)22-47	<0.01*
ALS-specific functions	60.46(13.68)30-92	78.68(9.85)49-96	<0.01*
Memory	14.01(4.80)0-23	15.60(3.89)2-23	0.07
Visuospatial	11.83(0.65)8-12	11.96(0.24)10-12	0.14
ALS-non-specific functions	25.85(4.94)12-35	27.56(3.93)14-35	0.06
Total ECAS score	86.31(16.51)50-122	106.24(12.16)68-128	<0.01*
ECAS time	18.13(3.85)11-30	18.77(3.21)13-28	0.09

Mean (SD) and range are reported forperformances in cognitive scores.

*p< 0.01.

### ECAS cut-off score and frequency of patients with cognitive impairments

The executive function was the most common domain, with approximately half of patients performing below the cut-off score, followed by fluency (20.24%) and language (15.48%). Approximately one-third of patients had cognitive impairments according to the total ECAS score (Table 4).

**Table 4:** Abnormal cut-off scores and frequency of patients with cognitive impairments.

Characteristics	Cut-off score	Patient No.	Percent
Total ECAS score	81.92	30	35.71
ALS-specific functions	58.98	36	42.86
ALS-non-specific functions	19.70	7	8.33
Executive	25.00	39	46.43
Fluency	11.48	17	20.24
Language	15.59	13	15.48
Memory	7.82	7	8.33
Visuospatial	11.48	6	7.14

Cut-off score: Mean-2SD.

### ECAS and other neuropsychological tests

Medium correlations were noted between the total ECAS score and FAB score as well as the scores of ALS-specific functions and FAB. Slight correlations were noted between the MMSE score and total ECAS score, ALS-specific functions score and separate ALS-non-specific functions score. No relationship was noted between the scores of ALS-non-specific functions and FAB (Table 5).

**Table 5:** Correlations between the scores of ECAS and other neuropsychological tests.

Characteristics	r	p
Total ECAS score& MMSE	0.48	<0.01*
Total ECAS score & FAB	0.52	<0.01*
ALS-specific functions & MMSE	0.42	<0.01*
ALS-specific functions & FAB	0.55	<0.01*
ALS-non-specific functions & MMSE	0.45	<0.01*
ALS-non-specific functions & FAB	0.29	0.01

r>0.8 implies high range correlation, 0.5<r<0.8implies middle range correlation, 0.3<r<0.5implies low range correlation, r<0.3 implies no relation.

*p< 0.01.

### ECAS and clinical features

The total ECAS score and education year exhibited a moderate correlation (r = 0.61, p<0.01). However, no obvious relationship was noted between the total ECAS score and age (r = -0.23, p = 0.04), ALSFRS-R (r = 0.28, p = 0.01). Although p<0.05 here, r<0.3 implied no relationship for correlation analysis. There was also no correlation between total ECAS score and duration of illness (r = 0.07, p = 0.54). No significant differences were found in the total ECAS scores between different sexes, diagnostic levels, sites of onset, and bulbar involvement (p>0.05).

### ECAS and education

Given that years of education and the total ECAS score were related, we divided the participants into a higher education group (>12 years) and a lower education group (≤12 years). No differences in age and gender were noted between the patients and controls of these two groups (p>0.05).

Interestingly, in the lower education group, significant differences were noted in memory (z = -1.99, p = 0.04) and ALS-non-specific functions (z = -2.15, p = 0.03). Language (t = -3.21, p<0.01), verbal fluency (z = -5.24, p<0.01), executive (t = -9.55, p<0.01), ALS-specific function (t = -9.54, p<0.01), and total ECAS score (t = -8.84, p<0.01) still differed between patients and controls; however, the visuospatial function did not (z = -1.50, p = 0.13).

However, in the higher education group, no differences were observed in language (t = -1.16, p = 0.25) and verbal fluency (t = -1.23, p = 0.23). Differences were noted in executive (t = -5.04, p<0.01), ALS-specific function (t = -4.46, p<0.01), and the total ECAS score (t = -4.31, p<0.01), whereas memory (z = -0.82, p = 0.41), visuospatial (z = 0.00, p = 1.00) and ALS-non-specific functions (z = -0.78, p = 0.44) did not differ.

### Frequency of patients with behavioural impairments

**Fig 1:**
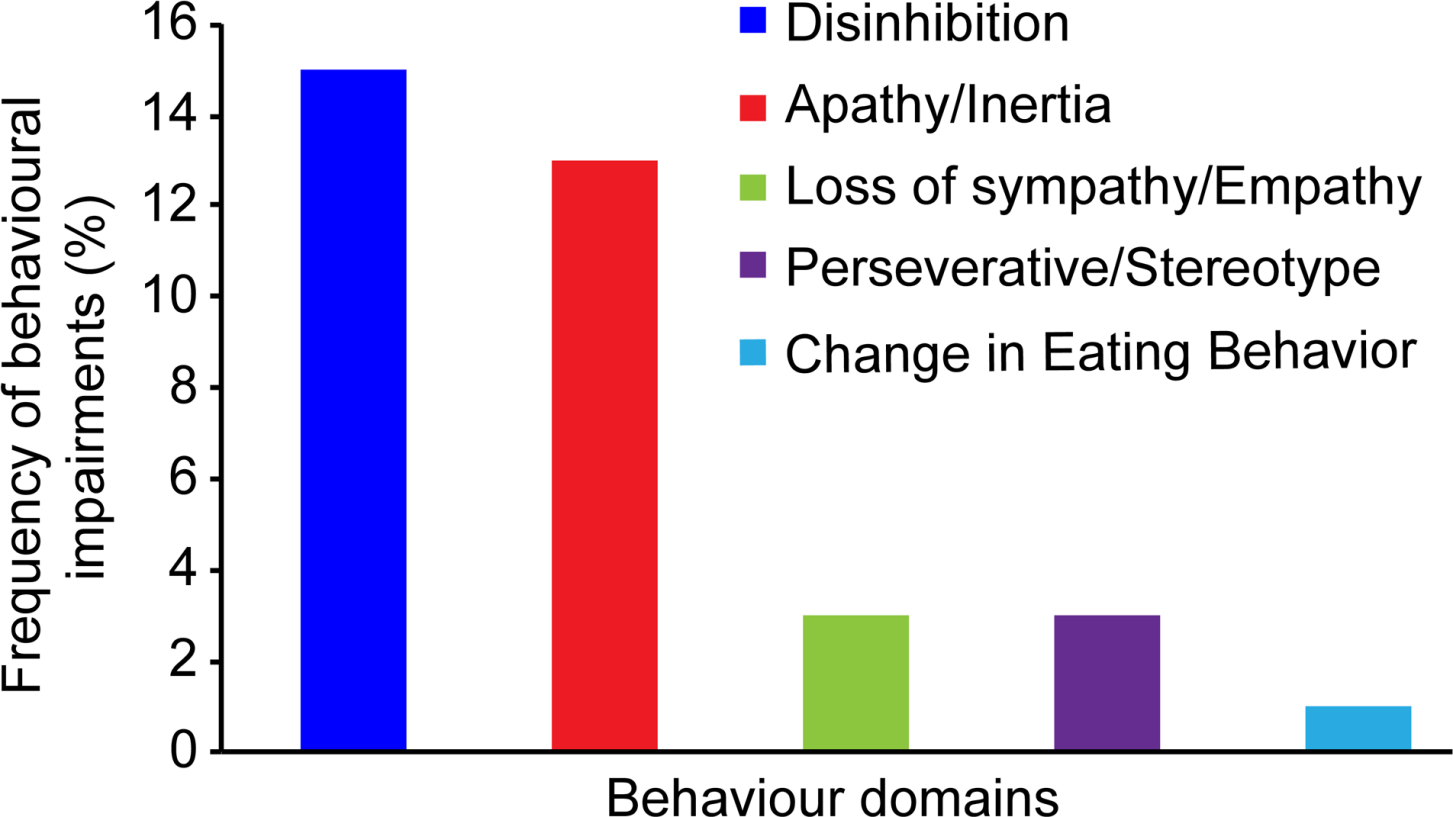
Behaviour Domains: frequency of behavioral impairments. 15 patients had disinhibition, 13 patients had apathy/inertia, 3 patients had loss of sympathy/empathy, 3 patients had perseverative/stereotype, and 1 patient had change in eating behavior.

The percentage of each domain is presented in Fig 1. One domain of behavioural impairment was observed in 14.28% of patients. In addition, 11.90% of patients had two domains, and 1.19% had three. Thus, 27.38% of patients exhibited behavioural abnormalities.

### Reliability

The Cronbach’s alpha coefficient of the Chinese ECAS version was 0.74, which was acceptable for exploratory research.

## Discussion

### Comparison between patients and controls

A significant difference in the total ECAS score was noted between patients and controls, indicating that this metric is potentially an index for cognitive assessment. We anticipated differences in the language, verbal fluency, executive function, and ALS-specific functions, whereas language was not as obvious as the others. Although language impairment was reported to be more common than executive function [10], no ALS language study has been specifically performed with the Chinese population. Spelling might be the largest difference between Chinese and English. Recent research has demonstrated that orthographic skills and morphological awareness were important contributors to Chinese reading and spelling development and that memory of these character stroke orders was important for character spelling in Chinese [33]. However, these functions might not be as sensitive as the executive function in ALS patients. However, the results provided a clue for further study of ALS language disorders in the Chinese population. Memory has also been shown to be affected in recent years [8,11]. Although the *p* values for memory and ALS-non-specific functions were >0.05, we noticed that it approached significance. It is worth mentioning that the average time for the ECAS was only approximately 18 minutes, which made it possible to use the Chinese version of the ECAS in the clinic as a routine test.

### ECAS cut-off score and frequency of patients with cognitive impairments

The cut-off value of the total ECAS score was 81.92 according to the method of Abrahams; 35.71% of patients performed below the value and were thought to have cognitive impairment. This percentage is consistent with previously reported data [2] and is slightly higher than 30.34%, which was observed by another Chinese group using ACE-R[28]. Notably, approximately half of patients exhibited executive impairment, which may cause the highly abnormal proportion of ALS-specific functions. According to unpublished data from our group, 20.0% of patients had this disorder when we screened 110 ALS patients and 96 controls for their executive functions [34]. Abnormality on more than two distinct tests that were sensitive to executive functions was used for diagnosing according to Strong’s criteria[1] in this screen. However, in the ECAS Chinese version, the executive category included digital span, alternation, sentence completion, and social recognition. Overlapping these four parts might enhance the sensitivity of the tool.

### ECAS and other neuropsychological tests

As FAB was designed especially for assessing executive function and ECAS is more focused on executive function, it is easy to understand why correlations existed between the total ECAS score, ALS-specific functions score and FAB score. The MMSE is a general scale that contains different domains of cognition. Thus, this screen could have a relationship with the total ECAS score, ALS-specific functions and ALS-non-specific functions score. However, the correlations might not be as strong as FAB score given lacking pertinence. ALS-non-specific functions included memory and visuospatial, whereas FAB was used for executive function. Thus, the scores of these tests were not closely related. Given that all the results could be explained, this might enhance the reliability of the Chinese version of the ECAS.

### ECAS and clinical features

We anticipated that education level would be correlated with the total ECAS score. However, gender [35] and age [25] were also thought to be important influential factors. One explanation might be that some other variants affect cognitive function, such as occupation and social economic status. However, we didn’t consider these factors in our research.

### ECAS and education

Interestingly, memory was more easily impaired in the lower education group, whereas verbal fluency and language function tended to be preserved in the higher education group. A cross-sectional and longitudinal study revealed that memory decline was faster in less-educated people [36].In the ALS population, this phenomenon might be similar. There was substantial evidence of an association between higher educational levels and a decreased incidence of dementia in various populations and studies. The main hypothesis was that clinical manifestations of cognitive disorders related to brain lesions were delayed in more highly educated people [37]. The preservation of brain lesions might minimize the differences in verbal fluency and language function in ALS patients. However, this hypothesis must be proven. As a general scale, ECAS is advantageous in testing different profiles of ALS cognitive impairments and could help to further explore the nature of the disease.

### Frequency of patients with behavioural impairments

The proportion of behavioural disorders was 27.38%. This result is slightly higher than our group’s previous study, which reported 20.9% with cut-off values from the Frontal Behavioural Inventory (FBI) and Neuropsychiatric Inventory (NPI) scores [34]. Only one patient exhibited impairment in three domains, which met the clinical features of FTD according to Neary’s criteria [38]. However, during the study period, there were another four ALS/FTD patients who had impairment in at least three behavioural domains. Since they had severe cognitive dysfunction, interfere with daily life and had abnormal MRI, fulfilling the dementia criteria of ICD-10, they were excluded from the 84 ALS patients when performing the cognitive test. If these four patients had been added, the FTD percentage would have reached 5.68%, which was in the range of 5 to 15% according to the literature [2,39].

### Reliability

In the Chinese ECAS version, the Cronbach’s alpha coefficient was 0.74. As an exploratory research, this was acceptable (a coefficient of 0.7–0.8 is considered good to excellent). The value was 0.77 in the English version.

## Conclusion

The Chinese ECAS version is the first general scale specifically designed for the Chinese ALS population. This test is an effective and rapid tool to screen cognitive and behavioural impairments in ALS patients. The Chinese ECAS may be more sensitive for assessing executive function and can help to further detect the nature of the disease. However, limitations of this research should be noted, including the lack of information about occupation, social economic status and inventories to evaluate a patient’s mood. Additionally, the Chinese ECAS version requires further validation regarding sensitivity and specificity.

## References

[1] Strong MJ, Grace GM, Freedman M, Lomen-Hoerth C, Woolley S, Goldstein LH, et al Consensus criteria for the diagnosis of frontotemporal cognitive and behavioural syndromes in amyotrophic lateral sclerosis. Amyotroph Lateral Scler. 2009;10: 131–146. 19462523 10.1080/17482960802654364

[2] Ringholz GM, Appel SH, Bradshaw M, Cooke NA, Mosnik DM, Schulz PE. Prevalence and patterns of cognitive impairment in sporadic ALS. Neurology. 2005;65: 586–590. 16116120 10.1212/01.wnl.0000172911.39167.b6

[3] Abrahams S, Leigh PN, Harvey A, Vythelingum GN, Grise D, Goldstein LH. Verbal fluency and executive dysfunction in amyotrophic lateral sclerosis (ALS). Neuropsychologia. 2000;38: 734–747. 10689049 10.1016/s0028-3932(99)00146-3

[4] Phukan J, Elamin M, Bede P, Jordan N, Gallagher L, Byrne S, et al The syndrome of cognitive impairment in amyotrophic lateral sclerosis: a population-based study. J Neurol Neurosurg Psychiatry. 2012;83: 102–108. 10.1136/jnnp-2011-300188 21836033

[5] Goldstein LH, Abrahams S. Changes in cognition and behaviour in amyotrophic lateral sclerosis: nature of impairment and implications for assessment. Lancet Neurol. 2013;12: 368–380. 10.1016/S1474-4422(13)70026-7 23518330

[6] Abrahams S. Executive dysfunction in ALS is not the whole story. J Neurol Neurosurg Psychiatry. 2013;84: 474–475. 10.1136/jnnp-2012-303851 23117493

[7] Ash S, Olm C, McMillan CT, Boller A, Irwin DJ, McCluskey L, et al Deficits in sentence expression in amyotrophic lateral sclerosis. Amyotroph Lateral Scler Frontotemporal Degener. 2015;16: 31–39. 10.3109/21678421.2014.974617 25482157 PMC4372458

[8] Machts J, Bittner V, Kasper E, Schuster C, Prudlo J, Abdulla S, et al Memory deficits in amyotrophic lateral sclerosis are not exclusively caused by executive dysfunction: a comparative neuropsychological study of amnestic mild cognitive impairment. BMC Neurosci. 2014;15: 83 10.1186/1471-2202-15-83 24981872 PMC4086690

[9] Mioshi E, Caga J, Lillo P, Hsieh S, Ramsey E, Devenney E, et al Neuropsychiatric changes precede classic motor symptoms in ALS and do not affect survival. Neurology. 2014;82: 149–155. 10.1212/WNL.0000000000000023 24336140

[10] Taylor LJ, Brown RG, Tsermentseli S, Al-Chalabi A, Shaw CE, Ellis CM, et al Is language impairment more common than executive dysfunction in amyotrophic lateral sclerosis? J Neurol Neurosurg Psychiatry. 2013;84: 494–498. 10.1136/jnnp-2012-303526 23033353

[11] Mantovan MC, Baggio L, Dalla BG, Smith P, Pegoraro E, Soraru' G, et al Memory deficits and retrieval processes in ALS. Eur J Neurol. 2003;10: 221–227. 12752394 10.1046/j.1468-1331.2003.00607.x

[12] Floris G, Borghero G, Chio A, Secchi L, Cannas A, Sardu C, et al Cognitive screening in patients with amyotrophic lateral sclerosis in early stages. Amyotroph Lateral Scler. 2012;13: 95–101. 10.3109/17482968.2011.605453 21895509

[13] Osborne RA, Sekhon R, Johnston W, Kalra S. Screening for frontal lobe and general cognitive impairment in patients with amyotrophic lateral sclerosis. J Neurol Sci. 2014;336: 191–196. 10.1016/j.jns.2013.10.038 24239183

[14] Lange F, Vogts MB, Seer C, Furkotter S, Abdulla S, Dengler R, et al Impaired set-shifting in amyotrophic lateral sclerosis: an event-related potential study of executive function. Neuropsychology. 2015. 10.1037/neu0000218 26167710

[15] Evans J, Olm C, McCluskey L, Elman L, Boller A, Moran E, et al Impaired cognitive flexibility in amyotrophic lateral sclerosis. Cogn Behav Neurol. 2015;28: 17–26. 10.1097/WNN.0000000000000049 25812127 PMC4375966

[16] Ji Y, Wei L, Chui D, Wang K, Fan D. Prospective memory tasks: a more sensitive method for screening cognitive impairment in ALS? BMC Neurol. 2012;12: 142 10.1186/1471-2377-12-142 23171421 PMC3551782

[17] Woolley SC, York MK, Moore DH, Strutt AM, Murphy J, Schulz PE, et al Detecting frontotemporal dysfunction in ALS: utility of the ALS Cognitive Behavioural Screen (ALS-CBS). Amyotroph Lateral Scler. 2010;11: 303–311. 10.3109/17482961003727954 20433413

[18] Gordon PH, Wang Y, Doorish C, Lewis M, Battista V, Mitsumoto H, et al A screening assessment of cognitive impairment in patients with ALS. Amyotroph Lateral Scler. 2007;8: 362–365. 17852014 10.1080/17482960701500817

[19] Simmons Z, Felgoise SH, Bremer BA, Walsh SM, Hufford DJ, Bromberg MB, et al The ALSSQOL: balancing physical and nonphysical factors in assessing quality of life in ALS. Neurology. 2006;67: 1659–1664. 17101900 10.1212/01.wnl.0000242887.79115.19

[20] van der Hulst EJ, Bak TH, Abrahams S. Impaired affective and cognitive theory of mind and behavioural change in amyotrophic lateral sclerosis. J Neurol Neurosurg Psychiatry. 2014;86: 1208–1215. 10.1136/jnnp-2014-309290 25476003

[21] Teng EL, Chui HC. The Modified Mini-Mental State (3MS) examination. J Clin Psychiatry. 1987;48: 314–318. 3611032

[22] Nasreddine ZS, Phillips NA, Bedirian V, Charbonneau S, Whitehead V, Collin I, et al The Montreal Cognitive Assessment, MoCA: a brief screening tool for mild cognitive impairment. J Am Geriatr Soc. 2005;53: 695–699. 15817019 10.1111/j.1532-5415.2005.53221.x

[23] Abrahams S, Newton J, Niven E, Foley J, Bak TH. Screening for cognition and behaviour changes in ALS. Amyotroph Lateral Scler Frontotemporal Degener. 2014;15: 9–14. 10.3109/21678421.2013.805784 23781974

[24] Niven E, Newton J, Foley J, Colville S, Swingler R, Chandran S, et al Validation of the Edinburgh Cognitive and Behavioural Amyotrophic Lateral Sclerosis Screen (ECAS): A cognitive tool for motor disorders. Amyotroph Lateral Scler Frontotemporal Degener. 2015;16: 172–179. 10.3109/21678421.2015.1030430 25967542

[25] Lule D, Burkhardt C, Abdulla S, Bohm S, Kollewe K, Uttner I, et al The Edinburgh Cognitive and Behavioural Amyotrophic Lateral Sclerosis Screen: a cross-sectional comparison of established screening tools in a German-Swiss population. Amyotroph Lateral Scler Frontotemporal Degener. 2015;16: 16–23. 10.3109/21678421.2014.959451 25292386

[26] Tran CD, Arredondo MM, Yoshida H. Differential effects of bilingualism and culture on early attention: a longitudinal study in the U.S., Argentina, and Vietnam. Front Psychol. 2015;6: 795 10.3389/fpsyg.2015.00795 26150793 PMC4471735

[27] Yuan Q, Jia J. Reduced cognitive function in Chinese patients with early amyotrophic lateral sclerosis and associated factors. Int J Neurosci. 2010;120: 641–646. 10.3109/00207454.2010.506585 20942578

[28] Wei Q, Chen X, Zheng Z, Huang R, Guo X, Cao B, et al Screening for cognitive impairment in a Chinese ALS population. Amyotroph Lateral Scler Frontotemporal Degener. 2015;16: 40–45. 10.3109/21678421.2014.966311 25309978

[29] Cui B, Cui L, Liu M, Li X, Ma J, Fang J, et al Neuropsychological investigation in chinese patients with progressive muscular atrophy. PLOS ONE. 2015;10: e128883. 10.1371/journal.pone.0128883 PMC4456153 26042930

[30] Brooks BR, Miller RG, Swash M, Munsat TL. El Escorial revisited: revised criteria for the diagnosis of amyotrophic lateral sclerosis. Amyotroph Lateral Scler Other Motor Neuron Disord. 2000;1: 293–299. 11464847 10.1080/146608200300079536

[31] Modern Chinese Frequency Dictionary: Beijing Language Institure Press, 1986.

[32] Dubois B, Slachevsky A, Litvan I, Pillon B. The FAB: a Frontal Assessment Battery at bedside. Neurology. 2000;55: 1621–1626. 11113214 10.1212/wnl.55.11.1621

[33] Yeung PS, Ho CS, Chan DW, Chung KK. Modeling the relationships between cognitive-linguistic skills and writing in Chinese among elementary grades students. Read Writ. 2013;26: 1195–1221. 23853420 10.1007/s11145-012-9411-6 PMC3695322

[34] Liu X, Fan D. Screen of cognitive and behavioural changes in Chinese Amytrophic Lateral Sclerosis patients. 2013.

[35] Palmieri A, Mento G, Calvo V, Querin G, D'Ascenzo C, Volpato C, et al Female gender doubles executive dysfunction risk in ALS: a case-control study in 165 patients. J Neurol Neurosurg Psychiatry. 2015;86: 574–579. 10.1136/jnnp-2014-307654 25063584

[36] Schmand B, Smit J, Lindeboom J, Smits C, Hooijer C, Jonker C, et al Low education is a genuine risk factor for accelerated memory decline and dementia. J Clin Epidemiol. 1997;50: 1025–1033. 9363037 10.1016/s0895-4356(97)00121-2

[37] Dufouil C, Alperovitch A, Tzourio C. Influence of education on the relationship between white matter lesions and cognition. Neurology. 2003;60: 831–836. 12629242 10.1212/01.wnl.0000049456.33231.96

[38] Neary D, Snowden JS, Gustafson L, Passant U, Stuss D, Black S, et al Frontotemporal lobar degeneration: a consensus on clinical diagnostic criteria. Neurology. 1998;51: 1546–1554. 9855500 10.1212/wnl.51.6.1546

[39] Bak TH, Hodges JR. Motor neurone disease, dementia and aphasia: coincidence, co-occurrence or continuum? J Neurol. 2001;248: 260–270. 11374089 10.1007/s004150170199

